# Recent developments in osteogenesis imperfecta

**DOI:** 10.12688/f1000research.6398.1

**Published:** 2015-09-07

**Authors:** Joseph L. Shaker, Carolyne Albert, Jessica Fritz, Gerald Harris

**Affiliations:** 1 Endocrinology, Medical College of Wisconsin, Milwaukee, WI, USA; 2 Orthopaedic and Rehabilitation Engineering Center, Marquette University and Medical College of Wisconsin, Milwaukee, WI, USA; 3 Shriners Hospitals for Children, Chicago, IL, USA

**Keywords:** Osteogenesis imperfecta, mutations, recessive

## Abstract

Osteogenesis imperfecta (OI) is an uncommon genetic bone disease associated with brittle bones and fractures in children and adults. Although OI is most commonly associated with mutations of the genes for type I collagen, many other genes (some associated with type I collagen processing) have now been identified. The genetics of OI and advances in our understanding of the biomechanical properties of OI bone are reviewed in this article. Treatment includes physiotherapy, fall prevention, and sometimes orthopedic procedures. In this brief review, we will also discuss current understanding of pharmacologic therapies for treatment of OI.

## Introduction

Osteogenesis imperfecta (OI) is an unusual heritable disease that occurs in about 1 in 10,000 to 20,000 live births ^1^. The major clinical manifestation is skeletal fragility. Skeletal deformity, joint laxity, and scoliosis may be present ^2^. Other extraskeletal manifestations include hearing loss, dentinogenesis imperfecta, blue/gray sclerae, hypercalciuria, aortic root dilatation, and neurologic conditions such as macrocephaly, hydrocephalus, and basilar invagination ^1– 5^. The phenotype is variable, ranging from osteoporosis presenting in adulthood to lethality in children ^3^. Even adults with “mild” OI may have significant musculoskeletal symptoms, including arthritis, fractures, back pain, scoliosis, and tendon ruptures ^6^.

About 90% of patients have mutations in type I collagen genes ( *COL1A1* and *COL1A2*) ^3^; however, many other genes have now been described. Some of the genes encode proteins related to type I collagen (for example, enzymes that modify type I collagen, chaperone proteins, and signaling proteins). In 1979, Sillence *et al*. proposed a classification system for OI with four types based on severity: type I mild non-deforming, type II perinatal lethal, type III severely deforming, and type IV moderately deforming ^7^. This classification has been expanded as new genes were discovered. Phenotypic classification (types I to V with multiple genes included in some of the types) has been proposed ^5^. Alternatively, classification by genetics has been proposed (see Table 1), which was created through modifications of references ^8– 10^.

**Table 1.** Classification of osteogenesis imperfecta.

Type	Inheritance	Gene	Protein	Defect	Phenotype
I	AD	*COLA1/COLA2*	α1(1) collagen	Collagen quantity	Mild, non-deforming
II	AD	*COLA1/COLA2*	α1(1)/α2(1) collagen	Collagen structure	Perinatal lethal
III	AD	*COLA1/COLA2*	α1(1)/α2(1) collagen	Collagen structure	Progressively deforming
IV	AD	*COLA1/COLA2*	α1(1)/α2(1) collagen	Collagen structure	Moderately deforming
V	AD	*IFITM5*	BRIL	Matrix mineralization	Moderate, distinct histology
VI	AR	*SERPINF1*	PEDF		Moderate to severe, distinct histology
VII	AR	*CRTAP*	CRTAP	Prolyl 3 hydroxylation	Severe to lethal
VIII	AR	*LEPRE1*	P3H1	Prolyl 3 hydroxylation	Severe to lethal
IX	AR	*PPIB*	CyPB	Prolyl 3 hydroxylation	Moderate to lethal
X	AR	*SERPINH1*	HSP47	Collagen chaperoning	Severe
XI	AR	*FKBP10*	FKBP65	Telopeptide hydroxylation	Progressively deforming (Bruck syndrome)
XII	AR	*SP7*	SP7/osterix	Osteoblast development	Moderate
XIII	AR	*BMP1*	BMP1/mTLD	Collagen processing	Severe, high bone mass
XIV	AR	*TMEM38B*	TRIC-B	Cation channel defect	Moderate to severe
XV	AR	*WNT1*	WNT1		Variable
XV	AD	*WNT1*	WNT1		Early-onset osteoporosis
Others					
	AR	*CREB3L1*	Oasis	COL1A1 transcription	Progressively deforming
	XL	*PLS3*	Plastin	Osteocyte defect	Mild
	AR	*PLOD2*	Lysyl hydroxylase 2	Collagen telopeptide hydroxylation	Progressively deforming

AD, autosomal dominant; AR, autosomal recessive; XL, x-linked.

There have been recent advances in the understanding of the structure and mechanical properties of bone in children with OI. These advances may lead to improved finite element (FE) models that help predict fracture risk of specific activities and help plan physiotherapy.

In addition to physiotherapy and orthopedic surgery when needed, intravenous bisphosphonates have been used extensively in moderate to severe OI in childhood. Less is known about pharmacologic treatment in adults. Anabolic therapy with PTH 1-34 has been studied in adults with OI. Future therapies may include antibodies to sclerostin, transforming growth factor beta (TGFβ) antagonism, gene therapy, and cell-based therapies.

## Genes and classification

OI is most commonly caused by mutations in type I collagen. Type I collagen is a rod-like structure formed from a trimer of 2 COL1A1 and 1 COL1A2 subunits ^3^, which requires post-translational modification. Many of the other rare forms of OI are due to defects in proteins involved in cross-linking, hydroxylation, and mineralization of type I collagen.

Mutations of *CRTAP*, which encodes cartilage-associated protein, have been shown to cause recessive OI ^11– 14^. Mutations of *LEPRE1*, which encodes prolyl 3 hydroxylase ^14– 16^, and *PPIB* (protein cyclophylin B) ^17– 19^ also cause recessive OI. The proteins described above form a complex that modifies specific prolines in the collagen and these mutations result in moderate to lethal OI.

*SERPINH1* mutations cause severe recessive OI ^20^. The protein affected in *SERPINH1* mutations, HSP47, is a collagen chaperone protein ^8^. *FKBP10* mutations cause recessive OI (progressively deforming) ^21^. This gene encodes the protein FKBP65, which appears to be needed for hydroxylation of collagen telopetide lysine ^22^. Both HSP47 and FKBP65 are needed for the proper folding of the collagen triple helix. Furthermore, Bruck syndrome (OI and congenital contractures) can be caused by homozygous mutations on FKPB10 ^23^, and Kuskokwim syndrome (congenital contractures with mild skeletal problems seen in Yup’ik people in Alaska) is caused by *FKBP10* mutations ^24^. *PLOD2* mutations also cause recessive OI ^25^. *PLOD-2* encodes lysyl hydroxylase 2, which hydroxylates collagen telopeptide lysine. Bruck syndrome can also be caused by homozygous mutations of *PLOD2* ^25^.

*BMP1* (bone morphogenetic protein 1) mutations also cause recessive OI ^26, 27^. The protein, BMP1, is a protease that cleaves the c-propeptide of type I collagen ^26, 27^ but also has other substrates. *SP7* mutations cause recessive OI ^28^. *SP7* encodes the protein osterix, which may be needed for osteoblast differentiation ^10^. *WNT1* mutations ^29– 31^ have been reported in early-onset osteoporosis (dominant) and OI (recessive). The protein, WNT1, may be important in the beta catenin system, which stimulates bone formation ^29– 31^.

*TMEM38B* mutations have been reported in recessive OI ^32^. This gene encodes TRIC-B, which may be important in intracellular calcium signaling. Defective TRIC-B may cause bone disease through defective calcium signaling in bone cells ^10^. *CREB3L1* mutations cause recessive OI ^33^. CREB3L1 encodes the protein OASIS, which may activate transcription of COL1A1 ^34^. *PLS3* (plastin 3) mutations have been reported in x-linked osteoporosis ^35– 37^. Plastin 3 is expressed in osteocyte dendrites and may be important in mechanosensing ^35^. Bone biopsies from patients with *PLS3* mutations have shown cortical and trabecular osteoporosis with normal to low bone formation rates ^36, 37^. There is no mineralization defect ^36, 37^.

Mutations in *IFITM5*, a bone-restricted IFITM-like protein (BRIL) (dominant) cause type V OI ^38– 42^. These patients have prominent callus formation and ossification of the forearm interosseous membrane ^38– 42^. They also have mesh-like lamellation on bone biopsy as well as a mineralization defect ^38– 42^. There appear to be substantial differences in phenotypic presentation even with similar mutations ^40– 42^. Type VI OI is caused by mutations in *SERPINF1* (protein PEDF) ^43, 44^. Children with type VI OI have elevated alkaline phosphatase, and bone biopsy reveals fish-scale pattern under polarized light as well as broad bands of unmineralized osteoid ^43, 44^. Interestingly, some patients with BRIL mutations have phenotypic type VI OI (rather than type V) ^45^. BRIL and PEDF are related, and it appears that mutations causing gain-of-function of BRIL cause OI type V and that those causing loss-of-function of BRIL look phenotypically like OI type VI ^46^.

## Structure and mechanical properties of bones in osteogenesis imperfecta

From a mechanical perspective, increased fracture risk in individuals with OI could stem from a combination of reduced bone mass, decreased bone material quality, and, in some individuals, the presence of bone deformity.

### Bone mass

Low bone mass is a clinical characteristic of OI, and individuals with this disorder tend to have markedly reduced areal bone mineral density (BMD) ^47– 49^. This reduced bone mass can be the consequence of decreased bone size or decreased volumetric BMD or both ^49, 50^. Studies of iliac crest biopsies have revealed lower bone tissue quantity in children with moderate and severe OI, including reduced bone volume fraction, and decreased trabecular and cortical thicknesses ^51– 53^. Decreased bone volume, though less marked, was also noted in some children with mild OI ^51, 52^.

In cortical bone specimens from the long bone shafts of children with OI, “atypical, flattened, and large resorption lacunae” ^54^ and abnormally elevated porosity have been observed ^54– 57^. For example, an average intracortical vascular porosity of 21% was found in bone shaft osteotomies from children with OI by synchrotron radiation micro-computed tomography ^55, 57^; the corresponding value in normal pediatric bones was 3% ^57^. From a structural perspective, reduced bone mass can lead to increased stresses within the bone as a result of a smaller area of bone tissue present to support physiological loads. For this reason, low bone mass is likely a considerable contributor to bone fragility in OI.

### Bone material quality

In addition to the structural deficiency (low bone mass), mechanical quality of the bone material in OI is reduced. The genetic defects causing OI affect type I collagen, the main organic component of bone. As discussed earlier, most forms of OI (types I to IV) are attributed to insufficient collagen production or amino acid substitution defects within the collagen molecules or both ^58– 63^, and less common recessive forms have been associated with abnormalities in other proteins that interact with type I collagen ^9, 64^. Since type I collagen is an integral component of bone tissues, it should be no surprise that abnormalities affecting this protein would impact bone material quality. At the ultrastructural level, irregularities in collagen and mineral geometry as well as abnormalities in mineral composition have been reported ^65– 70^. Studies in mice indicated that the material abnormalities in OI have a negative impact on bone material properties ^71– 76^. A few studies have also used biopsy and osteotomy specimens to measure bone material properties in humans with this disorder. Some of these studies used nanoindentation, a technique in which a diamond-tip indenter is pressed into the polished surface of a material (in this case, bone), creating an indent a few microns in size. With this test, elastic modulus and hardness—that is, properties representing the material’s resistance to elastic (recoverable) and plastic (non-recoverable) deformation, respectively—are determined at the submicrostructural level. Based on nanoindentation, slightly higher elastic modulus and hardness were found in children with mild (type I) versus severe (type III) OI ^77^, whereas these properties were not found to differ between children with severe (type III) versus moderately severe (type IV) phenotypes ^78^. However, exactly how these properties compare with normal tissues remains unclear; one study reported higher elastic modulus and hardness in children with severe OI versus controls ^79^, whereas another reported the opposite ^80^. Furthermore, bone tissues have a complex hierarchical structure, which results in properties that differ between length scales, and nanoindentation provides only limited insight regarding bone tissue properties at the submicrostructural scale. Another limitation with this technique is that it does not measure strength, a property representing the ability of a material to carry stress without breaking or sustaining damage.

Recent studies have measured cortical bone material properties, including strength, at a larger scale by using surgical bone specimens from long bone diaphyses of children with OI ^55, 56, 81^. In these studies, small osteotomy specimens were machined into parallelepiped-shaped specimens and loaded to failure in either bending ^55, 81^ or compression ^56^. Bone material strength was confirmed to be lower than normal in these children, and this property was found to be negatively related to an abnormally elevated intracortical porosity. These findings suggest that increased cortical porosity contributes to increased risk of long bone fractures in OI.

### Bone deformity

In addition to decreased bone mass and reduced bone material quality (low bone material strength), deformities of the spine and long bones are common in OI. For example, children with severe OI often exhibit anterolateral bowing of the femur and anterior bowing of the tibia ^7, 47^. Increased curvature in long bones leads to an increase in maximum stresses within the bone shaft ^82^. The increased stresses attributed to bone deformities in OI can further contribute to the risk of bone fracture.

### Fracture prediction based on mechanical models

Mechanical modeling through the use of FE analysis is a well-established technique that allows detailed analysis of composite structures under a variety of load conditions. In the field of orthopedic biomechanics, FE modeling is frequently used to examine the responses of bone to loading ^83– 86^. Patient-specific FE models have been effective for bone strain and fracture strength assessment, and as recently as 2009 Fritz *et al*. applied these models to predict fractures in OI ^87, 88^. A femoral model including muscle forces was analyzed during all seven phases of the gait cycle and geometrically matched to bone anatomy with x-rays. The most current work includes advanced meshing techniques for improved geometric biofidelity and updated mechanical property data ^55^. Other FE models for assessing OI bones have also been reported. Orwoll *et al*. used FE modeling to estimate vertebral strength in a study of the effects of teriparatide treatment in adults with OI ^89^. Caouette *et al*. developed an FE model to assess fracture risk at the tibia in children with OI ^90^. This tibia model examined fracture risk during two-legged hopping, lateral loading, and torsional loading. Future applications of FE modeling may prove invaluable for better quantification of fracture risk in OI. These models could help identify activities that pose greater risk of fracture and, through appropriate controls, may enable persons with OI to participate safely and more fully in a greater spectrum of daily and recreational activities.

## Management

### Physical therapy

The goals of the treatment in OI are to decrease pain and fractures and to maximize mobility. Physical therapy/rehabilitation ^91^ is particularly important in children to improve weight bearing and prevent fractures as well as to increase strength and mobility during fracture recovery. Some children may require wheelchairs or walking aids. Occupational therapy may be needed to help with daily living activities.

## Pharmacologic therapy

### Bisphosphonates

Bisphosphonates (BPs) are non-hydrolysable synthetic analogs of pyrophosphate ^92^. BPs adhere to mineralized surfaces, inhibit osteoclastic bone resorption, and have very long skeletal half-lives ^92^. Intravenous BPs are currently the primary treatment of children with moderate to severe OI. BPs increase BMD and size in children with OI ^49^. BPs do not appear to impair bone formation that increases cortical width in children with OI ^93^. Observational studies suggest decreased fractures ^94, 95^, decreased bone pain, and improved vertebral shape ^94, 95^. Ability to perform activities of daily living may also be improved. However, it has been difficult to confirm all of these benefits in randomized trials, and the optimal duration of BP treatment is unknown.

In a study of children with predominantly mild OI, oral risedronate increased BMD and appeared to decrease clinical fractures ^96^. Atypical fractures have been reported in children with OI treated with bisphosphonates ^97, 98^; however, osteneocrosis of the jaw does not appear to be a major problem in children with OI treated with BPs ^99– 101^. Several studies have been done on the use of intravenous or oral BPs in adults with OI. Although BMD increases have been reported during these treatments, fracture data are equivocal ^102– 106^. A Cochrane review found increased BMD in patients with OI treated with BPs but did not find definitive evidence of fracture reduction ^107^. Furthermore, a recent meta-analysis of placebo-controlled trials suggested that the effects of BPs for fracture prevention in OI were inconclusive ^108^.

### Growth hormone

Growth hormone has anabolic effects on bone. A 1-year randomized trial of the BP, neridronate, with or without growth hormone showed greater increase in BMD and growth velocity with growth hormone, but there was no fracture benefit of growth hormone ^109^.

### Teriparatide

Teriparatide (PTH1-34) is an anabolic agent that stimulates bone formation (and ultimately bone resorption). This drug decreases vertebral and non-vertebral fractures in post-menopausal women with osteoporosis ^110^. Observational data in adults with OI suggest increased BMD with teriparatide ^107, 111^. Recently, a randomized trial of teriparatide in adults with OI showed increased BMD as well as increased vertebral strength estimated by FE analysis ^91^. The benefits appeared to occur in mild (type I) OI but not in more severe OI (types III and IV).

### Denosumab

Denosumab is a monoclonal antibody to receptor activator of nuclear factor kappa B ligand that decreases bone resorption, increases bone density, and reduces fractures in women with post-menopausal osteoporosis ^112^. This drug may represent a future therapy in OI. In a study of four children with type VI OI, increased BMD and mobility and improved vertebral shape were reported after denosumab treatment, and the outcomes of this study indicated that this treatment appears to be safe ^113^. There is also a report of denosumab use in two children with OI caused by COL1A1/A2 mutations ^114^. As with BPs, “zebra lines” were present, suggesting continued longitudinal growth ^114^. Denosumab has been reported to cause hypophosphatemia, hypocalcemia, and secondary hyperparathyroidism in a child with fibrous dysplasia of bone ^115^. There was rebound hypercalcemia after stopping denosumab ^115^.

### Possible future therapies

Sclerostin is an inhibitor of the LRP5/WnT system that decreases bone formation. Antibodies to sclerostin are in clinical trials for treatment of osteoporosis with the goal to increase bone density ^116^. Sclerostin antibody appeared to be effective in a mouse model of moderately severe OI ^117, 118^ but less so in a mouse model of more severe OI ^119^. TGFβ is secreted by osteoblasts and increases osteoclastic bone resorption ^120^. Excessive TGFβ signaling may be important in some forms of OI, and anti-TGFβ therapy represents an interesting prospect for the future treatment of OI ^120^.

Cell-based therapy, such as bone marrow ^121^ or mesenchymal stem cell ^122– 124^ transplantation, has also been investigated and may have promise; but these could also have significant risks. Gene therapy with allele-specific silencing may represent a future therapy ^125^.

## Summary

Although most cases of OI are caused by COL1A1/A2 mutations, many new genetic causes have been identified in recent years. Some of these genes are related to the processing of type I collagen. Furthermore, we have greater understanding of the biomechanics of OI bone, including material properties, muscle and gait load effects, and fracture strength assessment. Biomechanical models could help identify activities that pose greater risk of fracture and, through appropriate controls, may enable persons with OI to participate safely and more fully in a greater spectrum of activities. Physical therapy is an important part of the management of these patients. Intravenous BPs are commonly used in children with moderate to severe OI. Some of the benefits seen in observational studies have been hard to prove in controlled studies. Treatment of adults with OI is less well studied. BPs and teriparatide appear to increase BMD, but fracture data are lacking. Teriparatide appears to increase bone strength as estimated by FE analysis in adults with mild OI. Other promising treatments for OI are under investigation.
