# Supplementary material for: Recent developments in osteogenesis imperfecta
Source: F1000Res. 2015 Sep 7;4(F1000 Faculty Rev):681. [Version 1] doi: 10.12688/f1000research.6398.1 (PMC4566283; doi:10.12688/f1000research.6398.1)
Supplement: Peer review history

# Peer review: Referee response for version 1

**DOI:** 10.5256/f1000research.6864.r10219

**Published:** 2015-09-07

**Authors:** Bart Clarke

**Affiliations:** 1 Division of Endocrinology, Diabetes, Metabolism, and Nutrition, Mayo Clinic College of Medicine, Rochester, MN, USA

I have read this submission. I believe that I have an appropriate level of expertise to confirm that it is of an acceptable scientific standard.

# Peer review: Referee response for version 1

**DOI:** 10.5256/f1000research.6864.r10218

**Published:** 2015-09-07

**Authors:** Dhanwada Sudhaker Rao

**Affiliations:** 1 Division of Endocrinology, Diabetes and Bone & Mineral Disorders, Henry Ford Hospital, Detroit, MI, USA

I have read this submission. I believe that I have an appropriate level of expertise to confirm that it is of an acceptable scientific standard.

# Peer review: Referee response for version 1

**DOI:** 10.5256/f1000research.6864.r10217

**Published:** 2015-09-07

**Authors:** Malachi McKenna

**Affiliations:** 1 St. Vincent's University Hospital, Dublin, Ireland

I have read this submission. I believe that I have an appropriate level of expertise to confirm that it is of an acceptable scientific standard.
